# Age-Related Evolution Patterns in Online Handwriting

**DOI:** 10.1155/2016/3246595

**Published:** 2016-09-26

**Authors:** Gabriel Marzinotto, José C. Rosales, Mounîm A. EL-Yacoubi, Sonia Garcia-Salicetti, Christian Kahindo, Hélène Kerhervé, Victoria Cristancho-Lacroix, Anne-Sophie Rigaud

**Affiliations:** ^1^SAMOVAR, Telecom SudParis, CNRS, University of Paris-Saclay, Palaiseau, France; ^2^AP-HP, Groupe Hospitalier Cochin Paris Centre, Hôpital Broca, Pôle Gérontologie, Paris, France; ^3^Université Paris Descartes, EA 4468, Paris, France

## Abstract

Characterizing age from handwriting (HW) has important applications, as it is key to distinguishing normal HW evolution with age from abnormal HW change, potentially triggered by neurodegenerative decline. We propose, in this work, an original approach for online HW style characterization based on a two-level clustering scheme. The first level generates writer-independent word clusters from raw spatial-dynamic HW information. At the second level, each writer's words are converted into a Bag of Prototype Words that is augmented by an interword stability measure. This two-level HW style representation is input to an unsupervised learning technique, aiming at uncovering HW style categories and their correlation with age. To assess the effectiveness of our approach, we propose information theoretic measures to quantify the gain on age information from each clustering layer. We have carried out extensive experiments on a large public online HW database, augmented by HW samples acquired at Broca Hospital in Paris from people mostly between 60 and 85 years old. Unlike previous works claiming that there is only one pattern of HW change with age, our study reveals three major aging HW styles, one specific to aged people and the two others shared by other age groups.

## 1. Introduction

Handwriting (HW) analysis has recently been investigated for detecting pathologies and cognitive decline [1–3]. In this context, age characterization from HW [4–6] is fundamental as it may allow distinguishing normal HW change due to age from abnormal one, potentially related to a cognitive decline. In this paper, we address the problem of age characterization from online HW. The goal is to detect HW styles and study their correlation with age, by the analysis of spatiotemporal HW parameters.

Several previous studies have tackled the problem of age characterization of healthy persons from both offline and online HW. Sometimes, this characterization is carried out by visual inspection [2, 3, 7–9] through observable features as, for example, letter size and width, slant, spacing, legibility or smoothness of execution, alignment of words with respect to baseline, and number of pen lifts. On the other hand, sometimes it is carried out by extracting automatically features from the offline raw signal [10] or from the raw temporal functions of online handwriting using a digitizer [4–6, 11, 12].

HW style characterization has been widely studied for both online [13] and offline [14] recognition tasks, and it is used to design writer style-dependent recognition models. Inference of HW styles, however, is difficult as there are no rules to define a HW style. A clustering algorithm is thus usually required (Gaussian Mixture Models [14], *K*-means [15], Self-Organizing Maps [13], Agglomerative Hierarchical Clustering [16], etc.). Previous works for clustering HW styles tackled the problem at the stroke level [16], character level [15], or word level [17]. We believe, however, that style characterization should rely not only on this raw signal information but also on high-level information associated with the variability observed across writer words.

Previous works on the correlation between age and HW agree that age leads to a different behavior of the features extracted from handwriting: change in the distribution of velocity profiles [5], increase of in-air time [6] and of the number of pen lifts [2], lower writing speed [4, 7, 18], lower pen pressure [2, 4, 7], irregular writing rhythm, irregular shapes of characters and slope [2], and loss of smoothness in the trajectory [2]. In most of such works, it is implicitly assumed that there is a unique pattern of handwriting evolution with age. Their analysis is mostly based on descriptive statistics (e.g., analysis of variance, linear regression).

We propose in this work to infer automatically different writing profiles and to study their correlation with age using unsupervised techniques. Our aim is to understand how handwriting evolves through age in terms of low-level information, namely, kinematic and spatial parameters extracted from handwritten words captured on a digitizer, and in terms of high-level information, characterized by stability measures across words. Since we have no* a priori* knowledge on how to define a HW style, we will use unsupervised techniques to automatically generate the HW styles that will later be analyzed under the scope of aging. Concretely, our unsupervised approach is based on a 2-layer clustering scheme that allows uncovering the main styles of online HW acquired on a digitizing tablet, with a special emphasis on elder HW styles. The 1st level separates HW words into writer-independent clusters according to raw spatial-dynamic HW information, such as slant, curvature, speed, acceleration, and jerk. The 2nd level operates at the writer level by converting the set of words of each writer into a Bag of 1st-Layer Clusters that is augmented by a multidimensional description of his/her writing stability across words. This 2nd-layer representation is input to another clustering algorithm that generates categories of writer styles along with their age distributions.

We have carried out extensive experiments on a large public online HW database covering all ages from teenagers to old people, augmented by HW samples of elders acquired at Broca Hospital in Paris. Thanks to this extended population, extra patterns of handwriting evolution emerge through age, with respect to our previous works in [19, 20]. Contrary to our previous works in [19, 20], an extensive study on such patterns through unsupervised learning techniques is here presented and a complete section is devoted to a new analysis on subjects older than 65 years.

The paper is organized as follows.  Section 2 presents the proposed approach including feature extraction, the two-level clustering scheme, and visualization techniques.  Section 3 describes the experiments and gives qualitative and quantitative assessments of our HW-based age characterization. Finally, in Section 4, the main conclusions are drawn and future directions are pointed out.

## 2. The Proposed Approach

In this section, we describe the feature extraction phase consisting of two stages, and we briefly describe the techniques we use to visualize HW features and the distribution of our multidimensional HW data.

### 2.1. Feature Extraction

Online HW words are described as a sequence of 3 temporal functions (*x*(*t*), *y*(*t*), *p*(*t*)) representing the pen trajectory and pressure on a digitizer [21]. At the 1st layer, we extract from each word 2 feature types. The first gathers local dynamic information, such as speed, acceleration, and jerk [16], while the second describes the static shape by measures such as stroke angles and curvatures [22] or intercharacter spaces [17]. As dynamic parameters, we consider horizontal and vertical speed computed locally at point *n* as *Vx* = |Δ*x*/Δ*t*| and *Vy* = |Δ*y*/Δ*t*| where Δ*x*(*n*) = *x*(*n* + 1) − *x*(*n* − 1), Δ*y*(*n*) = *y*(*n* + 1) − *y*(*n* − 1), and Δ*t*(*n*) = *t*(*n* + 1) − *t*(*n* − 1). These values are computed along the word and quantized to build 4-bin histograms over the *x*- and *y*-axes. We similarly compute local acceleration and jerk values, associated, respectively, with horizontal and vertical derivatives of speed and acceleration. In addition, pen pressure, its variation, and the pen-up duration ratio, computed as PR = (Pen-up Duration)/(Total Duration) [6], are considered, thus obtaining 33 global dynamic features. For spatial parameters, a resampling process is first performed to ensure that consecutive word points are equidistant, so that the parameter values at each point become equally representative, regardless of speed. Local direction and curvature angles are then extracted as in [22] and used to build 2 histograms of 8 bins quantized in the 0°–180° range. We also consider the number of pen-ups, the average horizontal in-air distance, the number of strokes (defined as writing movements between 2 local minima of speed), and their average length, as well as the average length of the stroke projections on *x*- and *y*-axes. This results in 21 spatial features. When combining dynamic and spatial features, a feature vector has dimension 54. At the 2nd layer, features are computed at the writer level. The writer's words are converted into a Bag of Prototype Words (BPW) [23] by assigning each word to its closest 1st-layer cluster and then generating the person's cluster frequency histogram. We add the histogram of intrawriter word distances by computing the Euclidean distance between the feature vectors of each possible pair of the person's words and quantizing these distances into a 5-bin histogram.

### 2.2. Unsupervised Approach: Clustering

HW style characterization is often approached using unsupervised techniques, such as clustering [13–15]. The reason to do so is that no* a priori* knowledge of the styles to characterize is available. These techniques, therefore, seek to cluster HW patterns that are similar into groups that appear naturally in the population and define the latter as styles. However, these HW styles characterizations are often carried out at the level of characters, strokes, and words [15–17], leaving aside the fact that writers may present some sort of variability in their styles across words. We consider this variability important to characterize HW styles. Therefore, we propose a 2-level approach: the 1st layer takes as input the dynamic and spatial parameters (low-level information extracted from the raw signal), while the 2nd layer studies the HW style variability of the writers (high-level information). At the first layer, we perform clustering of the set of words (using 54 features from Section 2.1) regardless of the identity of the writer, generating word clusters that characterize low-level styles. In the 2nd layer, the clustering is performed at the writer level, where each person is represented by his/her cluster frequency histogram and pairwise word distance histogram, in order to generate HW style categories that take into account the spatial and dynamic characteristics along with the writer's variability. We present the results carried out using *K*-means clustering on both layers (similar results are basically obtained with* GMM* or* hierarchical* clustering). To automatically determine the number of HW categories (clusters), we used the Silhouette criterion [24] as we do not have any* a priori* knowledge on the actual number of HW styles.

#### 2.2.1. Clustering Visualization

To visualize the quality of clustering, we use two dimensionality reduction techniques: Principal Component Analysis (PCA) and Stochastic Neighbor Embedding (SNE). PCA allows computing the correlations between features and the relevance of each for style characterization. SNE [25] is a nonlinear method that projects the points from a high dimensional space onto a new space preserving distance relations between points as much as possible.

#### 2.2.2. Clustering Quality Measures (Entropy Efficiency)

In order to objectively analyze the effects of the clustering on age characterization, we introduce three entropy efficiency measures [26]. These measures are not used to select the optimal number of clusters, but to evaluate the quality of the clustering once it is carried out. The first one quantifies the predictability of a certain age group (*A*_*i*_) distribution across the clusters and is computed using (1):

(1) ηAi=−∑k=1NCpCk ∣ Ailog2⁡pCk ∣ Ailog2⁡NC,

(2) ηCk=−∑i=1NApAi ∣ Cklog2⁡pAi ∣ Cklog2⁡NA,

(3) Eη=∑k=1NCCk⋃j=1NCCjηCk.

The second quantifies the degree of disorder of a cluster with respect to the distribution of the ages of the writers assigned to this cluster and is computed using (2). Finally, the third one gives a general measure of the quality of the whole clustering as a sum of the entropy efficiencies of each cluster, weighted by the size of the clusters as shown in (3). All the entropy efficiency measures are normalized between zero (maximum order → perfect age predictability) and one (maximum disorder → no possible distinction of age groups). In (1), (2), and (3), *C*_*i*_ stands for the *i*th cluster obtained in either the 1st or the 2nd layer; *A*_*i*_ corresponds to the *i*th age group (defined in Section 3.1); *N*_*A*_ is the number of age groups and *N*_*C*_ is the number of clusters.

## 3. Experiments

In this section, we describe our experiments including database description, the results obtained with the two clustering stages, the information theoretic measures we use to assess the effectiveness of our approach, and the results on the experiments run on the aged population only.

### 3.1. Database Description

For experiments, we use the IRONOFF database [27] of online HW word samples in English and French, acquired using a Wacom Tablet (UltraPadA4) that records a sequence of tuples (*x*(*t*), *y*(*t*), *p*(*t*)) sampled at 100 Hz with a resolution of 300 ppi. Although this database consists of 880 writers, only few are more than 60 years old (concretely 11 are between 60 and 77 years old). For a more reliable study of HW change as people age, we collected HW samples at Broca Hospital in Paris from a population of 25 persons with no diagnosed pathology, 23 of which are between 58 and 86 years old with an average of 72. These samples were also acquired on a Wacom Tablet (Intuos Pro Large) at the same sampling rate (100 Hz) but at a higher resolution (5080 ppi); we thus decreased the resolution of the new samples to match the 300 ppi of the IRONOFF database. Combining both databases, we obtain 27,683 HW samples from 905 writers from 11 to 86 years old (Y.O.). For age characterization, we split the obtained database into 6 age groups as shown in Table 1.

In this dataset seniors and elders are still underrepresented and age groups *A*_2_ and *A*_3_ are overrepresented. Therefore, to ensure meaningful results we balance the database, at the 2nd-layer stage, in terms of the age categories. To do so we proceed as follows: we divide the set of words written by a given person into groups from 10 to 15 words and assign each resulting group to a virtual new writer, making sure that the generated writers do not share words. Finally, to properly evaluate the clustering and its correlation with age, we retain the same number of virtual writers for each age group. This number was set to 26 writers per age group (thus generating a total of 156 writers), which were selected through *K*-medoids clustering over each *A*_*i*_ in order to retain the most representative writers of each age group.

### 3.2. Unsupervised Characterization of Age-Related Evolution Patterns in Handwriting

#### 3.2.1. First-Layer Clustering

**Figure 1:**
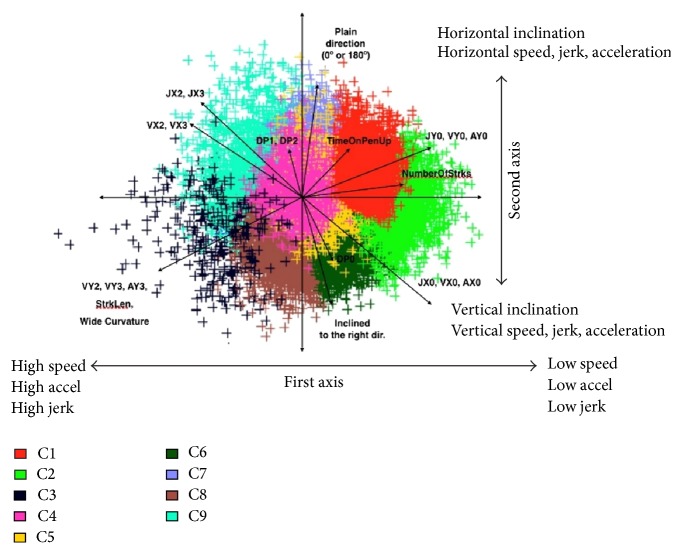
PCA projections of 1st-layer clusters over the first 2 principal components.

Using the Silhouette method, we observe that 9 is the optimal number of clusters for the 1st layer.  Figure 1 shows the 9 word clusters obtained by the *K*-means algorithm run over all the HW word samples, projected on the PCA plan spanned by the first two eigenvectors. As these two axes represent only 37% of the variance, some clusters overlap. Through PCA, we can represent the variables that have a larger contribution to the first two axes, also presented in Figure 1, and then attribute to each cluster particular characteristics with respect to the dynamic and spatial features. In particular, we observe that the main source of variability of the HW style is related to the dynamic features (speed, acceleration, and jerk) which appear highly correlated. The second main source of variation is the inclination of the HW, while in the third we find parameters such as pressure, pen-up time, and curvature.

**Figure 2:**
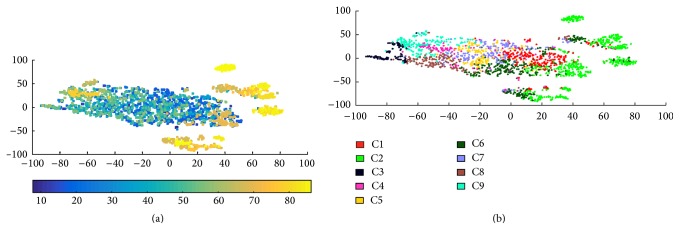
SNE projections of 1st-layer clusters: (a) age distribution; (b) 1st-layer clusters (the same colors in Figure 1 are used as identifiers of clusters).

As the first two principal components of PCA retain only 37% of overall data variance, one should be careful when interpreting the distribution of data over these two axes. This is why we considered also a visualization technique, namely, SNE, which is better at keeping the intrinsic information of data distribution in the high dimensional space. The SNE results, shown in Figure 2, were obtained on a subset of HW samples for better visualization. This subset is selected following two criteria: it is balanced in terms of the number of HW samples belonging to each age group and the representative samples of each age group are selected through *K*-medoid [28] clustering in order to retain most variability of the data.

In the SNE projections, we clearly observe that there is a correlation between age and HW, as it shows groups of old people that emerge automatically using our feature set. As seen from Figures 2(a) and 2(b), cluster 2 is mostly associated with aged people. We also note that clusters 3, 6, and 9 are partially associated with samples produced by aged writers.

**Figure 3:**
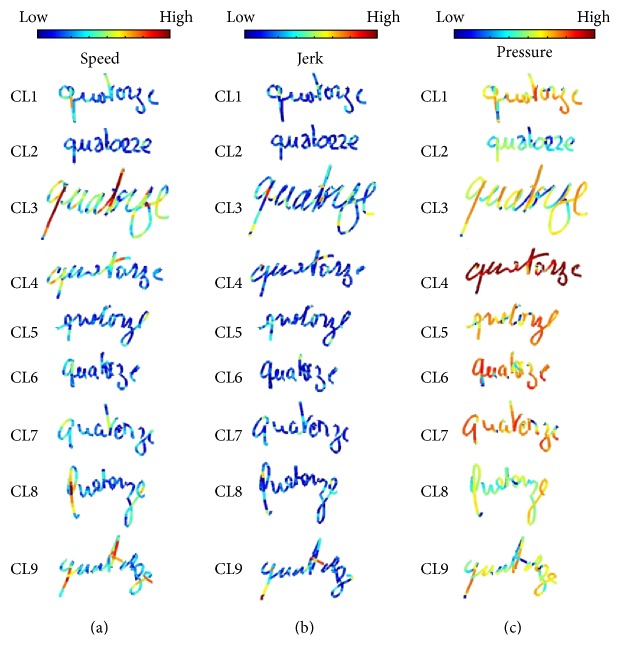
HW samples in each cluster in the 1st layer. The color scale quantifies the magnitude of speed (a), jerk (b), and pressure (c).

Figure 3 shows word samples in each cluster representing speed, jerk, and pressure in a color scale. From these word samples, we observe the main HW patterns that emerge from HW data. The characteristics of these patterns are summarized in Table 2.

If we study the samples corresponding to clusters 2, 3, 6, and 9 where we find HW samples from seniors (*A*_5_) and elders (*A*_6_), we observe that there are two main tendencies representing the aged population:

- Cluster 2 and cluster 6 represent small vertical script HW, with low speed, low jerk, and average pressure.
- Cluster 3 and cluster 9 represent a larger HW, which is cursive and inclined to the right, with very fast dynamics and average to low pressure.

#### 3.2.2. Second-Layer Clustering

**Figure 4:**
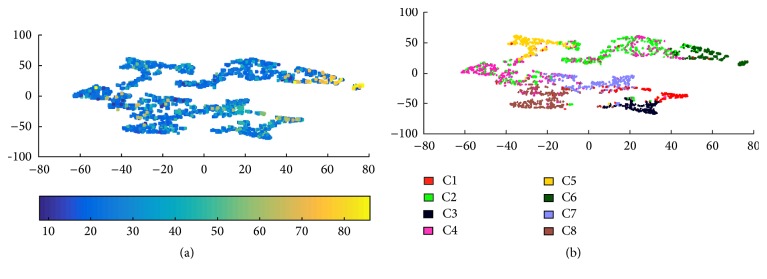
SNE projections of 2nd-layer categories: (a) age distribution and (b) 2nd-layer clusters.

At the first layer, we observe that it is complicated to analyze the cluster features as we detect the styles automatically, using a large set of parameters, which makes many combinations of them possible. This gives a new motivation to our second-layer clustering, looking forward to simplifying the results. At the second layer, the Silhouette method reveals 8 optimal categories.  Figure 4 shows the SNE projections of the 8 categories obtained by *K*-means run on the set of writers' 2nd-layer descriptors. In Figure 4, we also present the writers' age distribution. We remind the reader that in this layer each point represents a writer, described by 14 features:

- 9 features for the histogram of distribution of his words over the 1st-layer clusters,
- 5 features for his/her histogram of intrawriter word pairwise distances.

**Figure 5:**
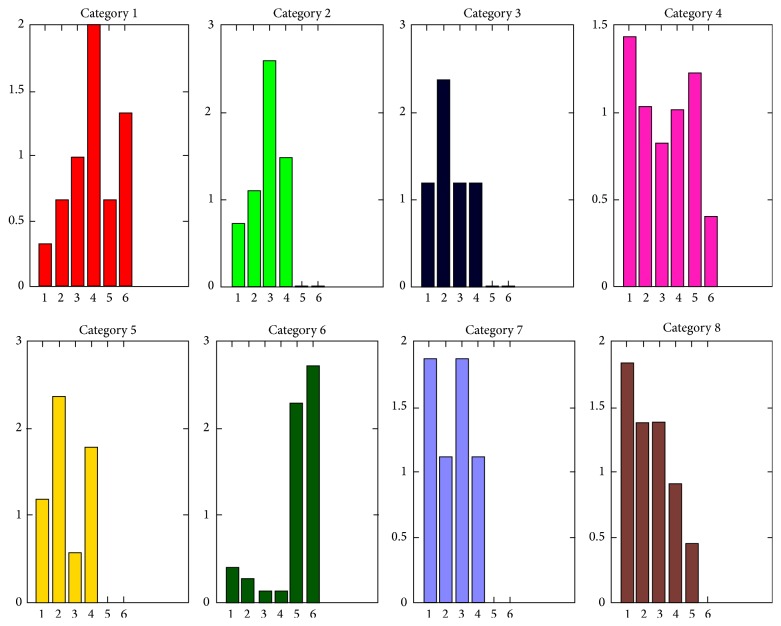
Age distribution in each category of the 2nd layer.

**Figure 6:**
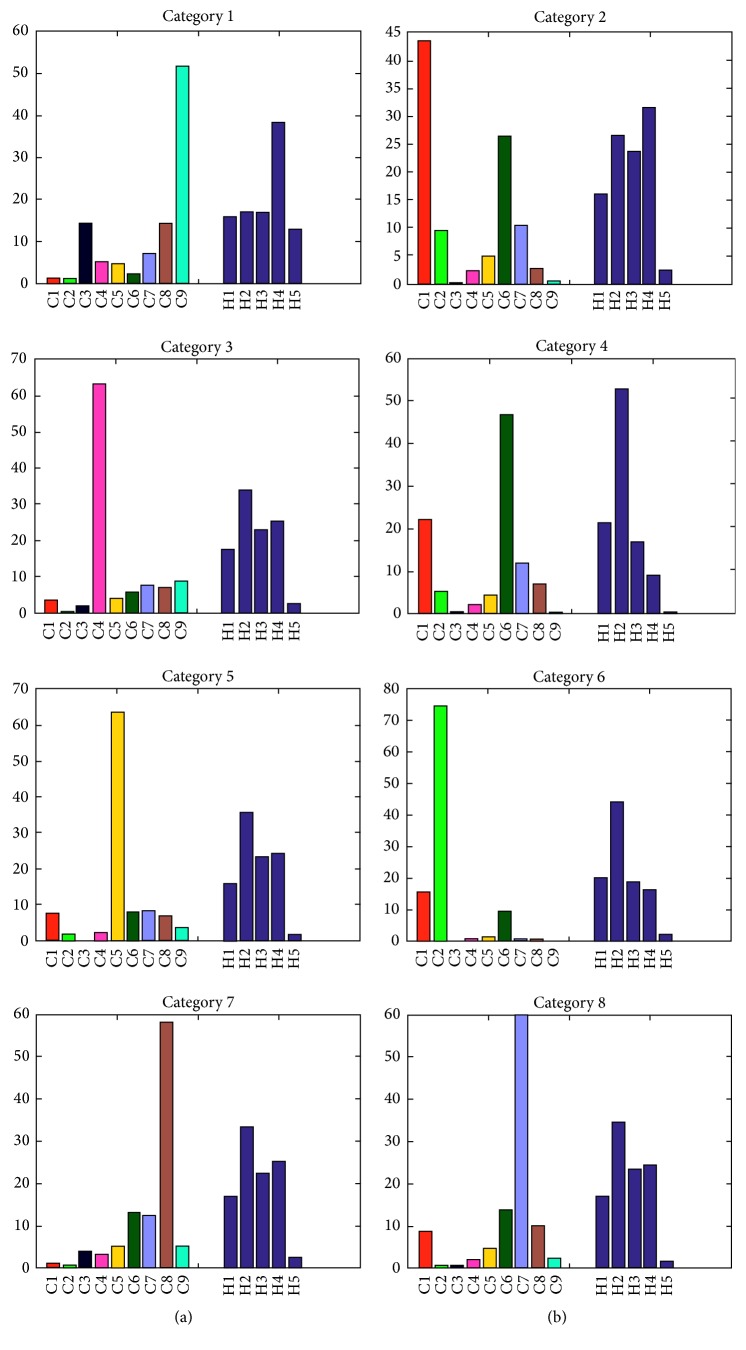
Representation of the distribution of 1st-layer clusters (a) and of pairwise distances between words (b) within each 2nd-layer category.

**Figure 7:**
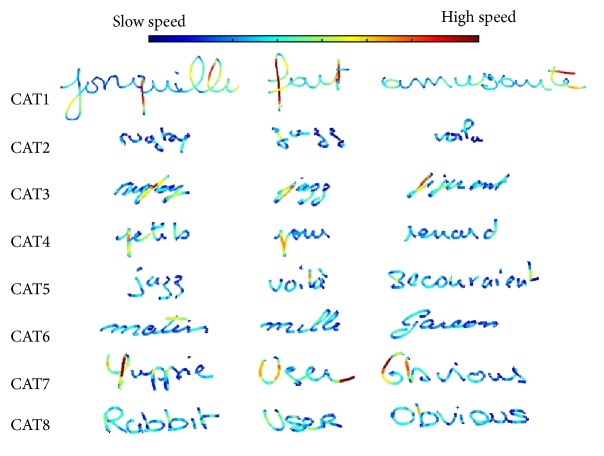
HW samples from each category of the 2nd layer showing speed on a color scale.

To study the correlation between HW styles and age, we analyze for each category the size of seniors' group (*A*_5_) and the size of elders' group (*A*_6_) with respect to the whole population within this category. The higher the percentage is, the more characteristic the category of the aged people is. As shown in Table 3, only 3 categories contain a significant amount of aged writers (these are categories 1, 4, and 6).  Figure 5 shows the age distribution for each category. The displayed histograms show the percentage of each age group in each category relatively to the initial balanced age distribution. For example, if age group *A*_4_ in category 1 takes value 2, this group is twice more represented in category 1 than in the balanced dataset.  Figure 6 shows the distribution of the center of each category in the 2nd layer over the 1st-layer clusters and the pairwise distance histogram, and Figure 7 shows some HW words of the most typical writer in each category (usually the writer whose representation is closest to the category center), when characterized by speed.

As we can see in Figure 5, category 6 gathers mostly persons above 65 years old. This category is the most stable, as writers maintain a relatively constant HW style across words. This category is also represented by cluster 2 in the 1st layer (as we can see in Figure 7) characterized by the lowest velocity, acceleration, and jerk, as well as very round HW with the highest number of strokes and smallest stroke length (as shown in the first layer's cluster characterization). As category 6 contains the highest number of persons (44 writers), this could indicate that the most common evolution pattern of aged persons is to develop a slow and curved HW with a medium to high “time on pen-up” (time in air) probably produced by hesitations when writing.

We also observe that category 1 contains a considerable quantity of persons aged above 75 years, as well as middle-aged individuals. This category is the one with the highest instability and is highly correlated with cluster 9 in the 1st layer, which is characterized by the highest velocity, acceleration, and jerk along with a low number of larger strokes. This could indicate the existence of a group of aged people that share with middle-aged people a more agile and fast HW, with tendency to produce long and straight strokes and a large style variation across words. In Figure 7 we can also observe how the writers in this category have a HW that is significantly larger.

Category 7 is also interesting since its age distribution contains all the age groups except the persons above 65 years old. This category is correlated with cluster 8 in the 1st-layer clustering stage. This group of people is characterized by high velocity, acceleration, and jerk in the vertical direction but an average value of these parameters in the horizontal axis, as well as high pressure during writing. Thus, this could indicate that other features that separate teenagers and middle-aged adults from the persons above 65 years old are a fast vertical HW with high *y*-axis velocities and jerk due to the upper and lower loops that represent high vertical stroke variance, but with an average velocity and jerk in the *x*-axis. Therefore, an average jerk and velocity in the horizontal axis could be an evidence of careful writing characterized by less variable strokes as the person writes in the horizontal sense, but at the same time, with high vertical velocity and acceleration to rapidly make the upper and lower loops.

Categories 4 and 8 are meaningful since they unveil differences between the eldest (*A*_6_) and the rest of the population. In this sense, category 4 consists of features that separate all the groups (*A*_1_–*A*_5_) from the eldest. On the other hand, category 8 contains fewer elders. Such an age distribution could indicate that the HW style consisting of average velocity, acceleration, and vertical jerk and low horizontal jerk is less frequent as age increases, thus characterizing the HW aging evolution. In other words, category 8 uncovers a typical, albeit nonfrequent, HW style of elders that consists of a low horizontal jerk even though speed, acceleration, and vertical jerk have average values. Categories 4 and 8 have very high and medium stability, and they are also correlated with clusters 6 and 7 in the 1st layer, respectively. This means that both categories have relatively low jerk in *x* with respect to velocity and acceleration, which is also the case for categories 2, 3, and 7 that do not contain any of the two elder groups (*A*_5_-*A*_6_). We also notice that category 4 has very low pressure variation and lower jerk on *x* compared to category 8 (which also has high pressure variation); thus, these elements could explain a very high stability for category 4 but no for category 8.

We also notice that category 3 in the 2nd layer, which has average instability, also contains all the age groups but the persons above 65 years old. This category is correlated with cluster 4 in the 1st layer, with the highest pressure and low jerk on the *x*-axis, as well as a lot of sharp HW turns. This could be an indicator, as we saw above in the analysis of category 7, that a low jerk on the horizontal direction and a relatively high HW pressure could separate the old people from the rest of the population.

Category 2 is another one that contains only persons from age groups *A*_1_ to *A*_4_, thus revealing other features that separate the elder persons from the teenagers and middle-aged groups. This category is related to clusters 1 and 6 in the 1st layer. Cluster 1 is characterized by low velocity and acceleration with average number of small strokes, average pressure, and average pressure variation. Cluster 6 consists of average velocities and acceleration and of an average number of pen-ups with short duration and an average number of strokes with average size. Both clusters share a very low horizontal jerk (that proved to be an important feature to separate elders from the rest of the population), an average pressure, and an average pressure variation.

Overall, we see that three different types of aged persons emerge based on their HW styles and stability:

- *Category 6.* This is the most frequent in elders and seniors (71.2%) and is associated with slow velocity and acceleration and a stable HW style, high time on air, and a large number of pen-ups. These characteristics are indicative of a slower and less fluent HW.
- *Category 1.* It represents 11.5% of old people and it consists of a HW style closer to that of middle-aged persons in terms of dynamic features. People in this group show the highest velocity, acceleration, and jerk, as well as a very high instability across words, which is the opposite behavior to category 6.
- *Category 4.* This is a new category of aged population emerging with respect to our previous works [19, 20]. It represents 15.4% of old writers and is characterized by a HW with average velocity, very low horizontal jerk, average pressure, low pressure variation, and high instability across words.

#### 3.2.3. Entropy Efficiency Measures

We measure the global entropy efficiency of the clustering as defined in (3) in terms of age distribution, on the balanced dataset with the same number of writers in the 6 age groups as described in Section 3.1. The reduction of entropy is used as a measure of how efficient the clustering is across layers in detecting HW styles that describe age tendencies. The result is shown in Table 4, where we can observe how the 2-layer approach reduces the entropy at each layer, which means that our clustering detects HW styles with different age distributions. Having a lower entropy efficiency in layer 2 than in layer 1 demonstrates that the stability of each writer HW style across words gives additional information for characterizing HW evolution through age.

Table 5 shows the entropy efficiency inside each of the categories of the 2nd layer as computed by (2). The lower the entropy efficiency, the more predictive the category of the writer's age. We observe that category 6 (mostly composed of elders) shows the lowest entropy, followed by categories 2, 3, 5, and 7, where no elders appear. This shows that these are the most interesting categories to analyze, in search for parameters which allow us to classify the elder population. In particular, one of the main findings is the HW style uncovered by category 6 which is the one that best predicts if the writer is an elder person. Likewise, the HW styles uncovered by categories 2, 3, 5, and 7 have good age prediction capabilities and in particular they rule out that the writer is an old person.

Finally, we also compute, using (1), the entropy of each age group with respect to the clusters on both layers. This allows us to detect which age groups introduce an entropy reduction for the clustering. The lower the entropy, the more predictable the age group of the clusters it will fall into, that is, the HW style or styles it will produce. The results of the cluster entropy efficiencies are shown in Table 6. We observe that the only age groups which introduce significant entropy reduction are *A*_5_ and *A*_6_, composed of people above 65 years old.

This entropy reduction validates our approach as it proves its capacity to characterize the HW of the elder population through few categories of writers and to discover a very limited set of different evolution patterns that the HW style exhibits as people grow old. On the other hand, observing almost no entropy reduction for age groups *A*_1_ to *A*_4_ implies that the HW style for these age groups shows a great variability across the population. Each person from 11 to 65 Y.O. can develop any HW pattern with a similar likelihood; in other words, there is no clear way to separate these age groups.

### 3.3. Clustering on the Aged Population

As observed in previous experiments [19, 20, 29], there appears to be more than one category that describes the HW of aged people. This confirms that there are several HW evolution patterns for aging, unlike previous findings in the literature which assume* a priori* that there is a single aging evolution pattern. In order to analyze in more detail the difference between these evolution patterns, we perform the two-layer clustering exclusively over the aged population (groups *A*_5_ and *A*_6_).

For the 1st layer, 3 clusters emerge when using *K*-means:

- *Cluster 1.* There are low speed, acceleration, and jerk (low dynamics), with average to high pressure and straight HW. This is the most common style of the aged population (75.6% of the samples); it is equally distributed over writers from 67 to 85 Y.O.
- *Cluster 2.* There are high speed, acceleration, and jerk, pressure above average, cursive, and almost no pen-ups. This style is less frequent (12.5%) and is observed in younger elders from 66 to 77 Y.O.
- *Cluster 3.* There is HW with very low speed, acceleration, and jerk, small size and long time on air, average to low pressure, and sharp angle breaks. It is observed only for few persons (11.9%) and is composed mostly of samples of people above 80 Y.O., that is, the most aged population.

**Figure 8:**
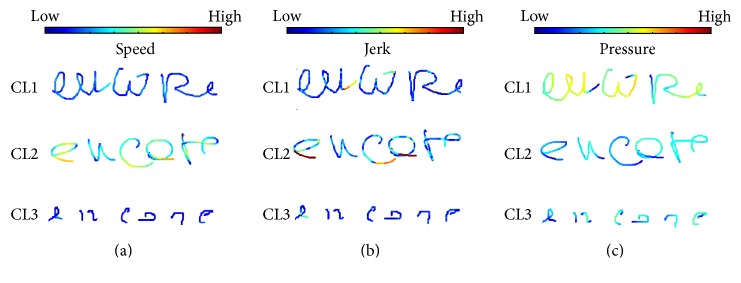
HW samples of the aged population in each cluster of the 1st layer. The color scale quantifies the magnitude of speed (a), jerk (b), and pressure (c).

**Figure 9:**
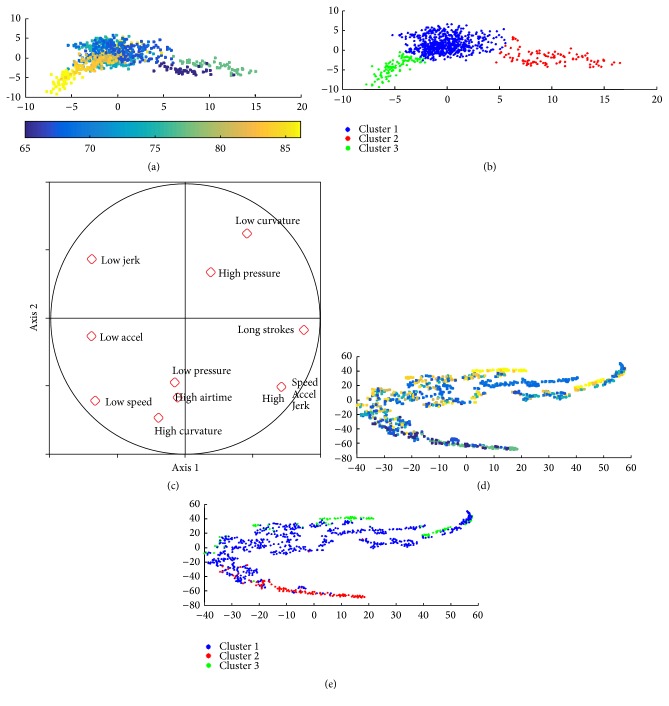
1st-layer clusters on the aged population: (a) PCA projections with age distribution (2 first axes; 43% of inertia); (b) PCA projections of the 1st-layer clusters, (c) correlation circle, (d) SNE projections with age distribution, and (e) SNE projections of 1st-layer clusters.

The results are presented in Figure 9 using PCA and SNE.  Table 7 summarizes the main characteristics of each group observed and Table 8 shows the age distribution in each cluster, in terms of average, standard deviation, minimum and maximum, and the width of the resulting age interval. Finally, Figure 8 displays some HW samples for each cluster.

At the 2nd layer, also 3 categories emerge through *K*-means:

- *Category 1. *There are same characteristics observed in cluster 1 (1st layer) and high stability across words. This HW style represents most of aged writers (76.2%), and it contains equally distributed writers from 67 to 85 Y.O. This category represents an average healthy aged writer. The low dynamics may indicate some loss of psychomotor skills, which can start as early as at 65 Y.O.
- *Category 2.* There are same characteristics observed in cluster 2 (1st layer) and low stability across words. This HW style represents 11.9% of the aged population. The writers therein are between 65 and 77 Y.O. We may hypothesize that these writers maintain most of their motor skills and are capable of executing complex motor tasks at high speed. That is why they show a larger instability as their HW changes across words. This category does not include any people above 80 years old.
- *Category 3.* There are same characteristics observed in cluster 3 (1st layer) and average stability across words. It contains 11.9% of the aged population. The writers therein are above 84 Y.O. These writers seem to have major problems to write, possibly due to a loss of psychomotor skills, vision problems, and so forth.

**Figure 10:**
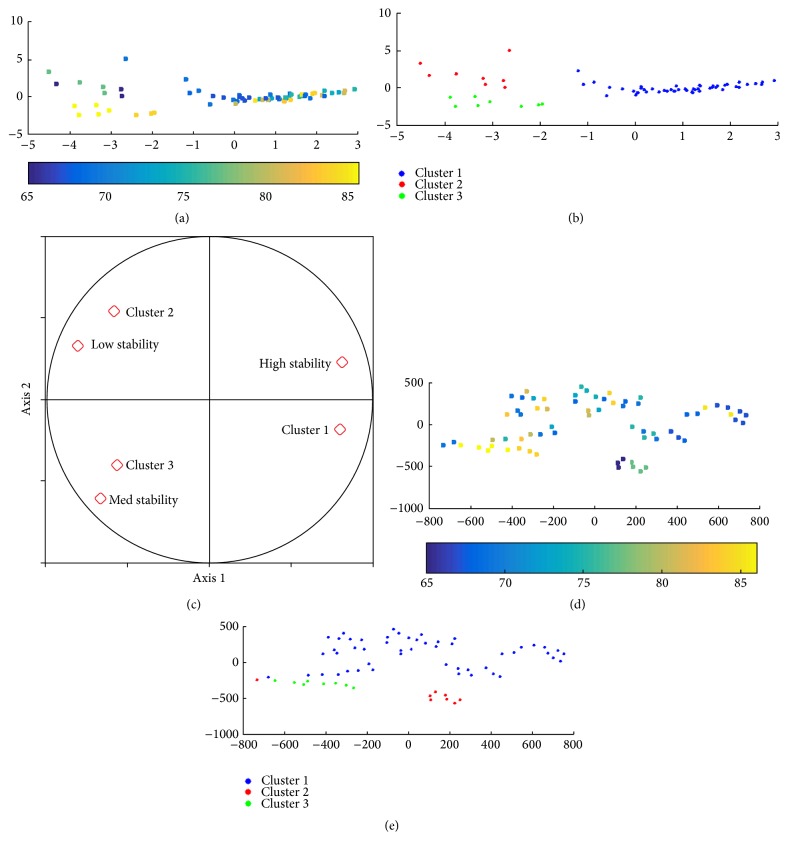
2nd-layer clustering on the aged population: (a) PCA projections with age distribution (2 first axes; 68% of inertia); (b) PCA projections of the 2nd-layer clusters; (c) correlation circle; (d) SNE projections with age distribution; (e) SNE projections of 2nd-layer clusters.

**Figure 11:**
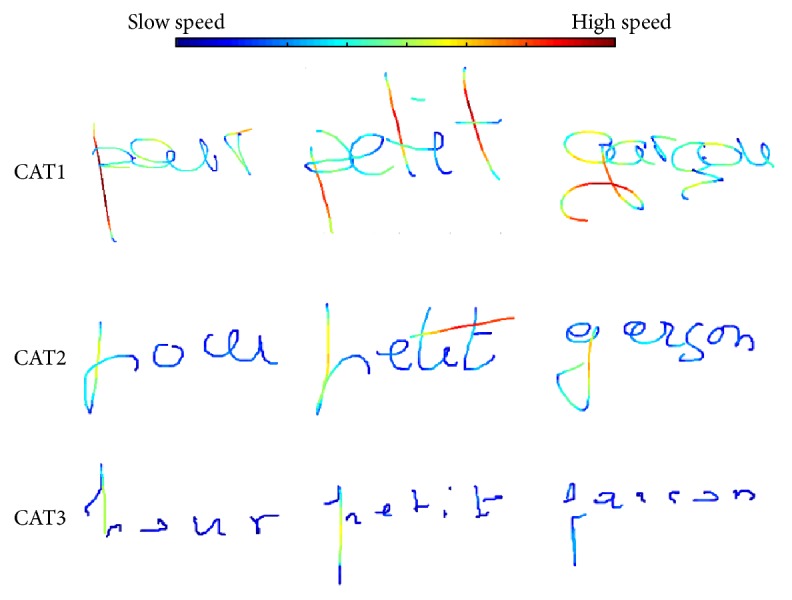
HW samples from each category of the 2nd layer showing speed on a color scale.

The results are presented in Figure 10. The statistics of the age distribution within each category are presented in Table 9, and some HW samples for these categories are shown in Figure 11.

This experiment confirms that aged people above 65 Y.O. present 3 HW patterns. These patterns vary mostly with respect to HW dynamics, pen pressure, and time on air. However, by observing the ages of the population inside these categories, we find that, above 80 years of age, people start sharing a rather unique style, with low dynamics (speed, acceleration, and jerk). The same thing cannot be said about HW stability across words: like HW dynamics, this parameter is a great source of variability across the aged population categories, but, above 80 years of age, it still does not show a particular trend on people's HW.

## 4. Conclusions and Perspectives 

We have proposed a novel approach for age characterization from online handwriting based on a 2-level scheme. The 1st level characterizes HW styles by raw spatial and dynamic information extracted from words and generates writer-independent word clusters. The 2nd level extracts, by contrast, the writer's HW style variability across words. This 2-layer representation is analyzed using unsupervised learning, for detecting relations between age and HW styles.

Our study has uncovered three different types of aged persons according to their HW styles and stability:

(i) The most important writing pattern in elders and seniors (category 6) is associated with slow velocity and acceleration and a stable HW style, consisting of high time on air and a large number of pen-ups, probably due to hesitations between strokes. This group, which is the most represented among the aged population (71.2%), has the highest number of smallest strokes. Overall, these characteristics are indicative of a slower and less fluent HW.
(ii) Some old people (11.5%) represented by category 1 have a HW style closer to that of a subset of middle-aged persons in terms of dynamic features. People in this group show the highest velocity, acceleration, and jerk, as well as a very high instability across words, which is the opposite behavior to the previously described writing pattern of category 6. They also present few and long strokes, which indicates a high fluency when writing. It is worth noticing that this writing pattern is overrepresented among elders (*A*_6_) with respect to seniors (*A*_5_). Indeed, there are some very aged persons that maintain handwriting skills.
(iii) Finally, a new category of elders emerges comparatively to our previous works [19, 20]. These are the old writers (*A*_5_ and *A*_6_) represented by category 4 (our second largest category) which are distinguished from a large part of the rest of population by a HW with average velocity, very low horizontal jerk, average pressure, low pressure variation, and high instability across words. It seems to be an intermediate writing pattern compared to the two previous ones and appears to represent 15.4% of the population.
(iv) There are about 28.8% of elders and seniors whose HW style cannot be distinguished from the average adult population. These aged writers are persons who maintained their skills as they aged, writing in a similar way to some parts of the adult population. From this skilled aged population, 60% are senior writers (*A*_5_) and 40% are elder writers (*A*_6_). This corroborates the tendency that the older the person gets, the more likely he/she will lose HW skills and fall into the group represented by category 6.

Another interesting finding by our approach is the fact that categories 2, 3, 5, and 7 do not contain any old person (*A*_5_ or *A*_6_). These categories disclose different HW styles of all the population except elders (*A*_6_) and seniors (*A*_5_). Categories 2 and 3 have average and low velocities and low and high stability, respectively, but they share a very low horizontal jerk with respect to speed and acceleration that is not present in the HW of the old population (the latter often features low jerk but this is explained by the fact that speed and acceleration are also low). Category 3 also has the highest pressure and low pressure variation, which seems to be other discriminative features between old people and the rest of the writers. As far as category 7 is concerned, it has average horizontal velocity, acceleration, and jerk and high vertical velocity, acceleration, and jerk, as well as a low number of long strokes (high fluency) and high pressure. This HW fluency has been shown to be another useful feature that discriminates part of the elders from the rest of the population. These results confirm what we obtained in our previous works [19, 20, 29].

Our additional experiment only based on the aged population confirms that people above 65 Y.O. present 3 HW patterns. These patterns vary mostly with respect to HW dynamics, pen pressure, and time on air. Above 80 years of age, however, people in general start sharing a rather unique style, with low dynamics (speed, acceleration, and jerk). HW stability across words, by contrast, does not show a particular trend on HW of people, even after 80 years of age.

Following this study, we are currently collecting a dataset of HW samples at Broca Hospital in Paris from elder people with Alzheimer and MCI cognitive disorders. Adding this population to the control population that served in this work, we will generalize our approach in order to assess its efficiency in automatically detecting HW styles associated with Alzheimer, MCI, and control persons. To do this, we will also explore supervised techniques to uncover HW features that are most characteristic of neurodegenerative decline.

## Figures and Tables

**Table 1:** Age groups.

Category	Age range	Number of writers
Teenagers (*A*_1_)	11–17 years	68
Young adults (*A*_2_)	18–35 years	639
Mid-age adults (*A*_3_)	36–50 years	133
Old adults (*A*_4_)	51–65 years	43
Seniors (*A*_5_)	66–75 years	14
Elders (*A*_6_)	76–86 years	8

**Table 2:** Main characteristics of first-layer clusters.

	Dynamics	Inclination	Pressure	Curvature	Pen-up
Cluster 1	Low speed, accel, jerk	Straight	Average	Round strokes	Average
Cluster 2	Very low speed, accel, jerk	Straight	Low	Round strokes	Many
Cluster 3	High speed, accel, jerk	Inclined right	Average	Straight strokes	Very few
Cluster 4	Average speed, accel, jerk	Inclined right	High	Straight strokes	Average
Cluster 5	Average speed, accel, jerk	Straight	Average	Average	Many
Cluster 6	Average dyn. on *Y*, low dyn. on *X*	Straight	Average	Average	Average
Cluster 7	Average speed, accel, jerk	Straight	Average	Round strokes	Average
Cluster 8	High dyn. on *Y*, average dyn. on *X*	Straight	Average	Straight strokes	Few
Cluster 9	Very high speed, accel, jerk	Inclined right	Average	Straight strokes	Average

**Table 3:** 2nd-layer categories: size and percentages of seniors (*A*_5_) and elders (*A*_6_).

	Cat1	Cat2	Cat3	Cat4	Cat5	Cat6	Cat7	Cat8
Size	18	16	10	29	10	44	16	13
Seniors (*A*_5_)	**11%**	0%	0%	**21%**	0%	**39%**	0%	6%
Elders (*A*_6_)	**22%**	0%	0%	**7%**	0%	**45%**	0%	0%

**Table 4:** Total entropy efficiency for each Layer.

	Layer 1	Layer 2
Entropy efficiency *E* [*η*]	0.8365	0.7935

**Table 5:** Entropy efficiency for each category in the 2nd layer.

	Cat1	Cat2	Cat3	Cat4	Cat5	Cat6	Cat7	Cat8
*η*(*C*_*K*_)	0.92	0.72	0.74	0.97	0.71	0.68	0.76	0.85

**Table 6:** Entropy efficiency for each age group within each layer.

	*A*_1_	*A*_2_	*A*_3_	*A*_4_	*A*_5_	*A*_6_
*η*(*A*_*K*_) layer 1	0.91	0.96	0.96	0.96	0.56	0.42
*η*(*A*_*K*_) layer 2	0.92	0.98	0.92	0.94	0.45	0.33

**Table 7:** Main characteristics of first-layer clusters for the aged people.

	PCT of people	Dynamics	Pressure	Curvature	Pen-up
Cluster 1	75.6%	Low speed, accel, jerk	Average to high	Straight strokes	Average
Cluster 2	12.5%	High speed, accel, jerk	Average to high	Straight strokes	Few
Cluster 3	11.9%	Very low speed, accel, jerk	Average to low	Sharp breaks	Many

**Table 8:** Age statistics in 1st-layer clusters obtained on the aged population.

	Number of samples	Average age	Standard dev. age	Min. age	Max. age	Width of age interval
Cluster 1	630 (75.6%)	73.61	6.31	67	85	18
Cluster 2	104 (12.5%)	71.00	5.84	65	77	12
Cluster 3	99 (11.9%)	84.43	3.24	69	86	17

**Table 9:** Age statistics in 2nd-layer categories obtained on the aged population.

	Number of samples	Average age	Standard dev. age	Min. age	Max. age	Width of age interval
Category 1	51 (76.2%)	73.45	6.26	67	85	18
Category 2	8 (11.9%)	71.50	6.02	65	77	12
Category 3	8 (11.9%)	85.25	1.04	84	86	2
